# Nano-Messengers of the Heart: Promising Theranostic Candidates for Cardiovascular Maladies

**DOI:** 10.3389/fphys.2022.895322

**Published:** 2022-07-11

**Authors:** Sneha Yedavilli, Anula Divyash Singh, Damini Singh, Rasmita Samal

**Affiliations:** ^1^ Department of Life Science, Central University of Karnataka, Kalaburagi, India; ^2^ Apollo Hospitals Educational and Research Foundation, Hyderabad, India; ^3^ Environmental Pollution Analysis Lab, Bhiwadi, India

**Keywords:** stem cell exosomes, myocardial repair, biomarker, biomimetic, therapeutic delivery

## Abstract

Till date, cardiovascular diseases remain a leading cause of morbidity and mortality across the globe. Several commonly used treatment methods are unable to offer safety from future complications and longevity to the patients. Therefore, better and more effective treatment measures are needed. A potential cutting-edge technology comprises stem cell-derived exosomes. These nanobodies secreted by cells are intended to transfer molecular cargo to other cells for the establishment of intercellular communication and homeostasis. They carry DNA, RNA, lipids, and proteins; many of these molecules are of diagnostic and therapeutic potential. Several stem cell exosomal derivatives have been found to mimic the cardioprotective attributes of their parent stem cells, thus holding the potential to act analogous to stem cell therapies. Their translational value remains high as they have minimal immunogenicity, toxicity, and teratogenicity. The current review highlights the potential of various stem cell exosomes in cardiac repair, emphasizing the recent advancements made in the development of cell-free therapeutics, particularly as biomarkers and as carriers of therapeutic molecules. With the use of genetic engineering and biomimetics, the field of exosome research for heart treatment is expected to solve various theranostic requirements in the field paving its way to the clinics.

## Introduction

The global prevalence of cardiovascular diseases (CVDs) has increased rapidly, accounting for nearly 18 million deaths per year and is expected to rise to over 23 million by 2030 (World Health Organization 2021). A compromised heart function results from myocardial damage caused by hypertension, atherosclerosis, heart attack, arrhythmia, or heart failure. Though heart transplantation is the gold standard treatment, the shortage of donor hearts and the risk of rejection are major limitations in treating CVD patients. Current treatment options can only manage end-stage heart disease using assisted devices (Capriotti and Micari 2019), hence there is a compelling need for effective treatment options. In the past decades, stem cells have been considered a safe and effective tool to improve the cardiac function of damaged hearts. However, clinical trials such as Allogenic Heart Stem Cells to Achieve Myocardial Regeneration (ALLSTAR) (Makkar et al., 2020), Cardiosphere-derived autologous stem cells to reverse ventricular dysfunction (CADUCEUS) (Makkar et al., 2012), demonstrated poor retention, low survival, and risk of arrhythmias in stem cell transplanted patients (Banerjee et al., 2018; Menasché 2018).

Exosomes, which are naturally formed nanocarriers (30–150 nm) of endocytic origin, play a crucial role in maintaining vascular integrity and cardiovascular health (Sahoo et al., 2021). They carry various biomolecules, e.g., proteins, lipids, RNA, DNA, long non-coding RNA (lncRNA), siRNA, etc., and transfer them to the target cells (Kowal et al., 2014). Owing to their role in maintaining cardiovascular health, exosomes, especially those secreted by stem cells, are considered attractive candidates for regenerative therapies (Sahoo and Losordo 2014). Unlike stem cells, exosomes derived from them are more stable during cryopreservation, are non-immunogenic, non-teratogenic and possess better retention potential post injection besides their ability to cross biological barriers (Marbán 2018). Hence, stem cell exosomes offer new hope in cell-free paracrine therapies for myocardial repair.

The therapeutic utility of exosomes against CVDs, especially those secreted by cardiac cells post-pathological insult, is being increasingly explored (Kishore and Khan, 2017; Saha et al., 2019). Understanding the characteristic properties of various cardiac cell-derived exosomes and their effects on cardiac function is one of the key areas of interest in CVDs (Rezaie et al., 2019). Stem cells of the human heart tend to secrete exosomes in response to injury, a part of which is also released into the circulation (Barile and Vassalli 2017), suggesting their crucial role as biomarkers for early diagnosis of CVDs. In this context, cardiac specific micro RNAs (miRNAs) present in exosomes have garnered huge attention (Zhou et al., 2018; Henning 2021). miRNAs are group of small non-coding RNAs of about 17–25 nucleotides, which act as post-transcriptional regulators of physiological processes (Wang J et al., 2016). Recent years have witnessed the implementation of various engineering strategies to develop exosome-based novel off-the-self therapeutics (Luan et al., 2017; Murphy et al., 2019). In the current review, we provide comprehensive information on stem cell-derived exosomes, their unique features, and highlight the mechanisms of their cardioprotective functions. We further discuss potential applications of exosomes as diagnostic and therapeutic tools in the treatment of CVDs, focusing on current advancements made in the field, including prospective future directions.

## Stem Cell-Derived Exosomes

**FIGURE 1:**
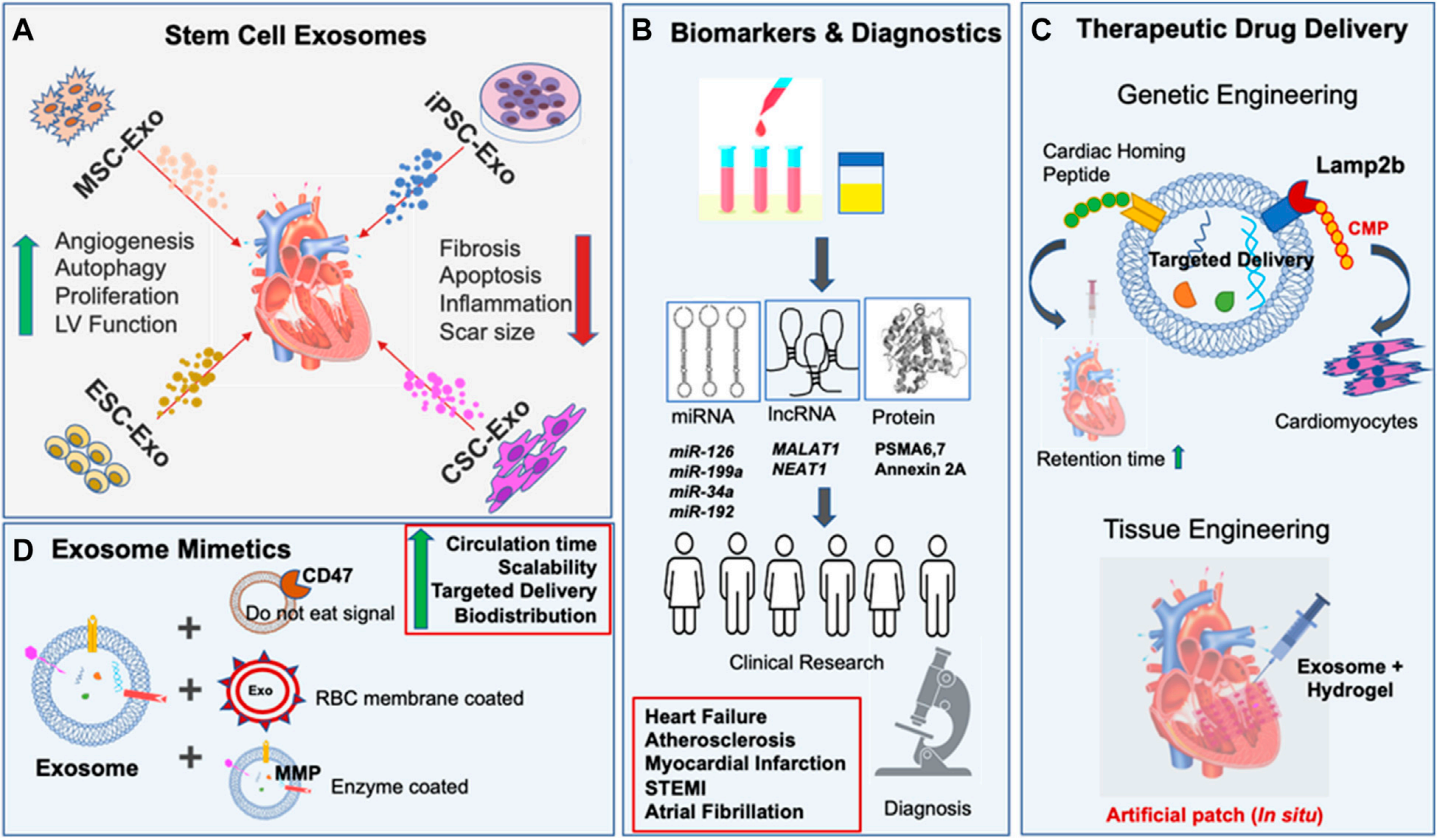
Role of stem cell exosomes in cardiovascular diseases. **(A)** Top panel indicates various stem cell exosomes involved in cardiac repair. The effective cardioprotective mechanisms are listed. Arrows indicate the regulation of various biological functions. **(B)** Schematic representation of applications of stem cell exosome as source of biomarkers for disease diagnosis. **(C)** Engineering strategies implemented to modulate the exosome properties are shown here. **(D)** Exosome-mimetics, designed using nanomedicine, enhance the therapeutic potential of natural exosomes.

Owing to their self-renewability and differentiation abilities, stem cells are promising candidates for regenerative therapies. However, the current focus on stem cells is being surpassed by exosomes derived from them, which are superior, safer, and scalable cell-free alternatives (Ong and Wu 2015). Stem cell-derived exosomes’ biogenesis and extraction methods have been reviewed in detail elsewhere (Li et al., 2017; Balbi and Vassalli 2020). In this section, we discuss the pleiotropic action of various stem cell-derived exosomes in regulating myocardial functions as illustrated in Figure 1A.

## Mesenchymal Stem Cell Exosomes

Mesenchymal stem cells (MSC) are multipotent in nature and possess great regenerative potential (Charles et al., 2020). They are isolated from various adult tissues, primarily bone marrow (BM), adipose tissue, and endometrium (Pittenger et al., 2019). Though the safety profile of MSC therapy is well known, its efficacy is not yet clear (Karantalis and Hare, 2015; Hare et al., 2017). Several preclinical and clinical studies confirmed improvement in cardiac functions post-MSC therapy (Razeghian-Jahromi et al., 2021), but clinical outcomes were variable (Perin et al., 2014). For example, a comparative study of three clinical trials showed variable effects of MSC therapy in DCM and ICM patients. While in DCM patients, MSC administration improved cardiac functional parameters, in ICM patients it induced cardiac remodeling (Tompkins et al., 2018). Over the past few years, MSC-derived exosomes (MSC-Exo) have gained increasing impetus. A comparative miRNA analysis of MSC and MSC-Exo revealed similarities in their miR expression profiles and superior cardioprotection offered by MSC-Exo treatment in rat MI model. Notably the expression of *miR-21* and *miR-15*, which are associated with hypertrophy and ischemic injury respectively were lower in MSC-Exo compared to MSC. The authors further demonstrated that MSC-Exo treatment *in vivo* markedly reduced inflammation, inhibited fibrosis, and improved cardiac function in MI rats (Shao et al., 2017). Consistently, MSC-Exo attenuated cardiac inflammation after ischemia/ reperfusion (I/R) injury in mice. Following exosome treatment for 3 weeks, IL6 levels were significantly reduced and IL10 was elevated in the heart tissue. In addition, miR182-mediated macrophage polarization was also observed by MSC-Exo *via miR182* (Zhao et al., 2019). The molecular cargo of MSC-Exo is modified in response to cardiac injury or in stressful conditions. For example, MSC-Exo enhanced H9C2 cell viability *in vitro* and reduced ROS production in an H2O2 stimulated myocardial ischemia reperfusion injury (MIR) injury rat model (Liu et al., 2017; Xiao et al., 2018). Interestingly, MSC-Exo regulated autophagy by reducing the levels of Bnip3 and LC3-II *via miR-125b-5p* in a murine MI model (Xiao et al., 2018). Exosomal cargo modulates autophagy in target cells to maintain cellular homeostasis in stressful conditions using different signaling pathways including Akt/mTOR, EGFR, and TLR signaling, etc. (Hassanpour et al., 2020).

A mechanistic insight into the adipose-derived MSC-Exo revealed Wnt/β-catenin mediated regulation of survival and apoptosis in cardiomyocyte (CM) and rat I/R-injury model (Cui et al., 2017). Recently the efficacy of MSC-Exo derived from ESC was reported in a preclinical study, where MSC-Exo when administered systemically in a porcine MI model *via* intravenous bolus injection for 7 days resulted in a significant reduction of the infarct size by 30%–40% and cardiac function was improved in comparison to control (Charles et al., 2020). Thus, MSC-Exo possesses immense potential to restore cardiac function.

## Embryonic Stem Cell Exosomes

Human embryonic stem cells (ESC), characterized by their unlimited self-renewal capacity and differentiability to any somatic cell type (Müller et al., 2018), are brilliant candidates for regenerative therapies. However, despite encouraging results, ethical challenges associated with the destruction of human embryos, immune rejection and safety issues concerning malignant transformations hamper their clinical use in regenerative therapies (Volarevic et al., 2018). Consequently, ESC-derived exosomes (ESC-Exo), which are the acellular derivatives of ESC, are being investigated (Kishore and Khan 2016). Molecular miRNA analysis revealed an enrichment of the *miR-290-295* cluster, particularly *miR-294* in murine ESC-Exo. Intramyocardial delivery of such exosomes reduced fibrosis, induced neovascularization, and augmented cardiac functions in mice following MI (Khan et al., 2015). Interestingly, human-ESC-derived cardiovascular progenitor cells extracellular vesicles including exosomes (ESC-Pg-Exo) reportedly improved ejection fraction, capillary density, and reduced infarct size after 6 weeks of treatment post-MI in a mouse model similar to their cellular counterpart (Kervadec et al., 2016). Similarly, human-derived ESC-Pg-Exo under hypoxia conditions promoted myocardial infarct healing by improving CM survival and angiogenesis, primarily regulated by lncRNA *MALAT1 via* targeting *miR-497* (Wu et al., 2020). Thus, ESC-Exo can act as a potent cell-free alternative to ESC, and further studies are desired in this context.

## Induced Pluripotent Stem Cell Exosome

The breakthrough discovery of Yamanaka factors’ having ability to reprogram somatic cells marks the descent of the era of induced pluripotent stem cells (iPSC). These cells are generated from reprogrammed adult somatic cells and have the ability to differentiate into any cardiac lineages (Lalit et al., 2014). In addition, they can be obtained from patients who allow autologous therapeutic options without any ethical concerns like ESC. However, tumorigenic potential, risk of arrhythmias and low retention rate have been the potential limitations in this area (Wang et al., 2015; Müller et al., 2018). Thus, iPSC-derived exosomes (iPSC-Exo) are being explored for precision medicine against CVDs (Yang 2018). From molecular analysis, iPSC-Exo was identified to express pluripotency-related markers like *Oct3/4A, Nanog,* and *Sox2*, similar to iPSC and miRNAs involved in angiogenesis, cell cycle regulation, and hypoxia adaption (Zhou et al., 2016; Adamiak et al., 2018). Previously, the miRNA and lncRNA profiles of human derived ESC-CM-Exo and iPSC-CM-Exo harvested under hypoxic and normoxic conditions were found to be similar including the expression of cardioprotective miRs like *mi-R1*, *miR-21*, and *miR-30* (Lee et al., 2017). A q-PCR analysis showed the abundance of *miR-21* and *miR-210* in iPSC-Exo, known for their cardioprotective roles by targeting programmed cell death protein 4 and Ephrin-A3, inhibiting apoptosis and promoting angiogenesis (Nazari-Shafti et al., 2020).

The safety and efficacy of iPSC-Exo have been reported in preclinical studies (Jung et al., 2017). Earlier, cardiac fibroblast (CF) derived iPSC-Exo protected H9C2 CM from H2O2-induced oxidative stress by suppressing Caspase 3/7 activation. Intramyocardial injection of iPSC-Exo also exerted an anti-apoptotic effect in the MIR injury mouse model (Wang et al., 2015). Another comparative analysis of exosomes of iPSC derived from CF and dermal fibroblast of Heart Failure (HF) patients revealed lower expression of *miR22 in* iPSC-Exo, reflecting a loss of hypertrophy memory and attenuating cardiac remodeling. Further iPSC-Exo treatment significantly improved the differentiation of embryoid bodies to CM *in vitro* (Kurtzwald-Josefson et al., 2020)*.* Similarly, iPSC-derived extracellular vesicles including exosomes were found cytoprotective by inducing angiogenic, migratory, and anti-apoptotic properties in cardiac endothelial cells *in vitro*. The authors further reported an improved left ventricle (LV) function of the infarcted heart when iPSC-Exo was injected intramyocardially in MI mice. While iPSC injections led to teratoma formation, iPSC-Exo was found to be safer (Adamiak et al., 2018).

In addition, exosomes from iPSC-derived cells are considered a promising source for autologous delivery of cardiac specific bioactive contents (Jung et al., 2017). Notably, iPSC-derived cardiomyocytes (iCM) exosomes (iCM-Exo) were found to mimic iCM as they contain CM specific cargo (Santoso et al., 2020). In this study, iCM-Exo enhanced the CM’s survival in hypoxic conditions *in vitro* and improved cardiac function in a murine MI model. Also, iCM-Exo upregulated autophagy and autophagic flux during hypoxia-ischemia stress. In another study, human iPSC-derived cardiac cell (iPSC-CC) exosomes protected iCM by reducing apoptosis, maintaining calcium homeostasis and increasing energy metabolism. *In vivo,* they improved myocardial function, wall stress, and cardiac hypertrophy of the porcine infarcted heart without increasing arrhythmogenic frequency (Gao et al., 2020). However, a recent comparison of efficiacy of exosome isolated from iPSC-derived cardiovascular progenitor cells (iPSC-Pg-Exo) and iCM-Exo revealed superiority of iPSC-Pg-Exo in improving the survival, proliferation of CMs *in vitro* and also promoting angiogenesis of HUVECs in a dose-dependent manner. Further iPSC-Pg-Exo improved cardiac function by significantly enhancing Left ventricle ejection fraction (LVEF) and reducing left ventricular volumes in comparison to iCM, 7 weeks after exosomes treatment to chronic HF mouse model (El Harane et al., 2018). Thus, exosomes from iPSC and iPSC-derived cells hold great potential for the development of patient-specific regenerative therapies against CVDs.

## Cardiac Stem Cell Exosome (CSC-Exo)

Cardiac stem cells residing in the heart are unique owing to their origin, cardio-specific cargo, and inherent regenerative capacity (Messina et al., 2004). These multipotent cells resident in the human heart are clonogenic, have a defined cardiac fate, and can differentiate into cardiac-committed cells, which are affected by their ambience in pathological settings (Khaksar et al., 2018). The regenerative potential of human cardiac progenitor cells (CPC) and cardiosphere-derived cells (CDC), which are harvested from human tissue specimens, is well known from earlier clinical trials (Makkar et al., 2012). However, poor survival and poor functional integration of transplanted cells restricted their clinical translation (Gnecchi et al., 2008). Hence, the therapeutic potency of exosomal-derivatives of cardiac cells is gaining major attention for cardiac repair and regeneration (Sahoo and Losordo 2014).

CPC-derived exosomes (CPC-Exo) recapitulate the therapeutic benefits of CPC (Müller et al., 2005). The cardioprotective ability of patient-derived CPC-Exo was found superior to BM-Exo by preventing CM apoptosis *in vitro* and improving ventricular function in I/R rats, which was mediated by PAPP-A enriched in CPC-Exo (Barile et al., 2018). In another study, CPC-Exo released under hypoxic conditions showed enhanced proangiogenic and antifibrotic activity *in vitro* and improved heart function in an I/R injury model (Gray et al., 2015). Interestingly, CPC-Exo treatment nullified the Doxorubicin/Trastuzumab (cancer drug)-driven cardiotoxicity by attenuating fibrosis and iNOS expression (Milano et al., 2020). Similarly, mouse heart derived CPC-Exo protected CM from oxidative stress in I/R injury mouse exhibiting an anti-apoptotic effect mediated by *miR-451* (Chen et al., 2013). During viral myocarditis H9C2 infected with coxsackievirus B3, rat-derived CPC-Exo exhibited anti-apoptosis by inducing Bcl-2 activity and inhibiting caspase-3 and 9 cleavages (Li et al., 2019). Thus, CPC-Exo is packed with potent regulators for restoration of the damaged heart. Likewise, CDC exosome (CDC-Exo) mimicked the cardio-regenerative ability of CDC (Li et al., 2012). A number of preclinical studies pointed towards significant improvement in heart function following CDC-Exo treatment. For example, human CDC-Exo induced regeneration of the infarcted heart by promoting CM survival and angiogenesis. Following CDC-Exo treatment, cardiac function improved in MI mice, primarily mediated by *miR-146a* (Ibrahim et al., 2014). Recently, a randomized preclinical study in a porcine MI model revealed improved cardiac function, reduced scar-size, and an antifibrotic effect of CDC-Exo (Gallet et al., 2017). Further, the authors elucidated that the efficacy of CDC-Exo was notable when delivered intramyocardially, while intracoronary delivery was ineffective. Consistently, CDC treatment resulted in increased neovascularization, myocyte proliferation, and reduced fibrosis that was abolished by blunting the exosomal release, suggesting possible CDC-Exo mediated myocardial improvement (Hirai et al., 2020). Additionally, the immunomodulatory role of CDC-Exo was reported to be superior to MSC-Exo in a comparative study, where CDC-Exo polarized M1 macrophages to an M2-like phenotype in a mouse MI model, reducing inflammation (Walravens et al., 2021). Following CDC-Exo treatment, cardiac function was improved 4 weeks post-MI by reducing scar size and increasing infarct wall thickness. Thus, CDC-Exo is a promising therapeutic candidate for treating CVDs.

CMs, the chief cell type of the heart, play a crucial role in the cardiac conduction system. CM-derived exosomes (CM-Exo) are enriched in heat shock proteins (Hsp20, Hsp60, Hsp70) attributed to CM growth and survival under stress (Gupta and Knowlton, 2007). For example, elevation of Hsp20 in CM promoted exosome secretion, CM^Hsp^-Exo imparted cardioprotection by inducing angiogenesis in hyperglycemic stress conditions *in vitro* and attenuated diabetes-induced cardiac dysfunction and remodeling (Wang L. et al., 2018). Similarly, hypoxia-induced CM-Exo promoted apoptosis and inhibited proliferation, migration, and invasion of CF *in vitro* and *in vivo via* lncRNA AK139128 (Wang and Zhang 2020). Interestingly, glucose-deprived conditions induced secretion of CM-Exo loaded with glucose transporters and glycolytic enzymes, which mediated crosstalk between EC, suggesting metabolic regulation (Garcia et al., 2016). Additionally, ischemia-induced CM-Exo contains high amounts of MMP9 and MMP2, which are responsible for ECM degradation and tissue remodeling (Ribeiro-Rodrigues et al., 2017). Apart from the beneficial effects of CM-Exo, detrimental effects are also reported. For example, CM-Exo mediated adverse remodeling of the heart by inducing cardiac fibrosis *via miR-208a*. CM-Exo were enriched in *miR-208a* in hypoxic conditions, which was transferred to CF, promoting their proliferation and differentiation into myofibroblasts *in vitro*. Inhibition of *miR-208a* reduced cardiac fibrosis and improved cardiac function in MI rats (Yang et al., 2018). Thus, CM-Exo plays a major role in cardiac remodeling.

Taken together, all these observations suggest that stem cell exosomes offer immense potential to heal the wounded heart, as illustrated in Figure 1A. A summary of the cardioprotective mechanisms exhibited by stem cell exosomes from various *in vitro* and *in vivo* studies is listed in Table 1.

**Table 1:** Cardioprotective mechanisms of stem cell exosomes. A summary of various mechanisms involving stem cell exosomes in cardiac repair identified using *in vitro* and *in vivo* models.

Source	Biological effects of stem cell exosome		Reference
	*In vitro*	*In vivo*	
Bone marrow- derived MSC	Enhanced proliferation and inhibited apoptosis of H9C2 cells; inhibited fibroblast transformation into myofibroblast in BJ fibroblast cells by reducing α-SMA expression	7 days post-MI, MSC-Exo treatment increased LVEF and FS and reduced fibrosis and inflammation in MI rat model	Shao et al. (2017)
	Mediated macrophage polarization from M1 to M2 phenotype and reduced inflammation in RAW264.7 cells	Reduced infarct size, preserved LVESV/LVEDV, reduced fibrosis and hypertrophy of CM, and decreased IL-6 levels in serum and heart; transformed M1 macrophages to M2 phenotype in the mouse I/R injury model	Zhao et al. (2019)
	Increased cardiomyocyte autophagy *via* AMPK/mTOR and Akt/mTOR signaling; reduced cell apoptosis and ROS production in H9C2 cardiomyocytes	Reduced apoptosis and infarct size, upregulated LC3B expression and improved ejection fraction and FS in the MIR injury rat model	Liu et al. (2017)
	MSC-Exo reduced autophagic flux and enhanced cell viability of neonatal mouse CMs *via* miR-15-5p; downregulated p53/Bnip3 signaling	MSC-Exo exerted anti-autophagic effect *via* miR-125-5p, reduced infarct size, and improved ventricular remodelling in MI mice	Xiao et al. (2018)
Adipose-derived MSC	Reduced hypoxia/reoxygenation, induced myocardial apoptosis, and enhanced myocardial viability of H9c2 cells *via* Wnt/β-catenin signaling	ADMSC-Exo-antagonized I/R injury induced myocardial necrosis, and apoptosis in rats mediated *via* Wnt/β-catenin signaling; reduced infarct area	Cui et al. (2017)
ESC-derived MSC		Reduced infarct size by 30%–40%; preserved wall thickness; reduced transmurality of infarct zone in the porcine MI model after 7 days of intravenous injection	Charles et al. (2020)
Embryonic stem cell (murine)	Enhanced proliferation, survival, and cardiac commitment in CPC under H2O2 challenge	Promoted myocyte survival and proliferation *vi*a miR-294-3p, increased EF and FS, and reduced scar size in mice 8 weeks post-MI injury	Khan et al. (2015)
ESC-derived cardiovascular progenitor (ESC-Pg-Exo) (Human)	Hypoxia-conditioned ESC-Pg-Exo promoted angiogenesis of EC and improved survival of CMs mediated *vi*a *LncRNA MALAT1*	Intramyocardial delivery of Exo post-MI reduced scar size, improved CM survival in MI mice, and mediated *via LncRNA MALAT1*	Wu et al. (2020)
		Reduced LVESV and LVEDV and increased vascular density and contractile function 6 weeks post treatment in MI mice	Kervadec et al. (2016)
iPSC	Enhance cardiac EC migration, angiogenesis, and survival	Improve LV function in MI mice, reduce interstitial fibrosis, reduce myocyte apoptosis and enhance angiogenesis	Adamiak et al. (2018)
iPSC (Human CF)	Protects against H_2_O_2_-induced oxidative stress by inhibiting caspase 3/7 activation in H9C2 cells	Antiapoptotic effect in MI mice by supressing caspase 3 protein expression and protected cardiomyocytes from apoptosis	Wang et al. (2015)
	Improved differentiation of embryoid bodies to CM	—	Kurtzwald-Josefson et al. (2020)
iPSC-CM	Enhanced CM survival in hypoxia and promoted autophagy and autophagic flux in hypoxic cardiomyocytes	Reduces apoptosis and fibrosis in murine MI model and autophagy and autophagic flux was upregulated in the peri-infarct region	Santoso et al. (2020)
	Promote tube formation, maintain intracellular Ca^2+^ homeostasis, reduce apoptosis (hypoxia induced), and increase ATP levels in HUVECs	Improve LVEF, myocardial cellular energy metabolism, angiogenesis, and apoptosis; reduce scar size and cardiac hypertrophy in the porcine MI model	Gao et al. (2020)
iPSC-Pg	Improved survival of H9c2, proliferation of H9c2 cell and ESC-CMs, and induced angiogenesis of HUVECs	Improved LVEF and reduced LVESV and LVEDV 7 weeks after exosomes were injected to the chronic heart failure mouse model	El Harane et al. (2018)
CPC (rats)	Reduced coxsackievirus B3 (CVB3)-induced apoptosis of H9C2 cells and decreased expression of viral capsid protein 1 and pro-apoptotic factors of Bim/Caspase families	Prevents CM apoptosis *via* inhibition of cleavage of caspase-3 and caspase-9 in the CVB3-induced myocarditis model and reduces CVB3 replication by regulating the Akt/mTOR pathway	Li et al. (2019)
CPC (murine)	Protects CM from H_2_O_2_-induced apoptosis by decreasing caspase 3/7 activity, enhances tube formation of EC, and decreases pro-fibrotic gene expression in TGF beta-stimulated fibroblas**t**	Inhibited CM apoptosis mediated by miR-451 in I/R mice	Chen et al. (2013)
CPC (Human)	Inhibits CM death induced by hypoxia and glucose deprivation. Prevents Staurosporine-induced death and apoptosis in HL-1 cells *via* PAPP-A. Stimulates tube formation in EC	Improved LVEF; reduced scar size *via* PAPP-A expression; increased blood vessel density in the infarct region of the rat IR model and permanent coronary ligation model	Barile et al. (2018)
	Hypoxia-induced CPC enhanced angiogenesis of EC and downregulation of fibrotic gene expression	12 h hypoxia induced exosomes, reduced fibrosis, and improved FS in I/R rats	Gray et al. (2015)
	Reduced dox-induced oxidative stress and apoptosis in rat CM	Prevents myocardial fibrosis and LV dysfunction; inhibited iNOS production and CD68+ inflammatory infiltrates in Dox/Trz-treated rats	Milano et al. (2020)
Human CDC	Enhanced CM proliferation and survival and increased angiogenesis in HUVECs	Improved heart function in MI mice, decreased scar mass, increased viable tissue *via* miR-146a, and lower proinflammatory cytokine levels	Ibrahim et al. (2014)
	—	Improved systolic function and LVEF, increased viable mass, decreased scar size, and induced proliferation of CM in the porcine MI model	Gallet et al. (2017)
	Polarized M1 macrophage to M2 phenotype and upregulated Arg1/Nos2 ratio in peritoneal macrophages	Reduced scar size; increased infarct wall thickness in and murine MI model, showed a decrease in peritoneal macrophages in the mouse model of acute peritonitis	Walravens et al. (2021)
Porcine CDC	Reduce TNF-α-induced cardiac apoptosis and inflammation *via* miR-146a-5p in human cardiomyocytes	Improved heart function, increased neovascularization and cardiomyocyte proliferatio,n and reduced fibrosis in the porcine model of DCM	Hirai et al. (2020)
CM (mouse)	Hsp20 overexpressing-Exo improved mouse cardiac EC proliferation and migration under hyperglycemic conditions. Exerts anti-oxidative effect by lowering levels of ROS	Hsp20-overexpressing-Exo increased the density of myocardial blood vessel, inhibited cardiac apoptosis, and adverse remodelling in diabetic mice	Wang X. et al., 2016
CM (rats)	Ischemia induced CM-Exo promote proliferation, angiogenesis of EC, enhance secretion of MMP by EC and promote ECM degradation	Ischemic exosomes improve neovascularization, increased survival in MI mice	Ribeiro-Rodrigues et al. (2017)
	Hypoxic CM-Exo lowers cell viability and migration and enhanced apoptotic activities of rat CFs *via* AK139128	CFs displayed higher apoptotic levels with increased level of Bcl-2 while decreased expression of Bax, reduced migration and invasion in the MI rat model	Wang and Zhang (2020)
	Exosomes from glucose-depleted CM exerted increased glucose uptake and pyruvate production and diffusion in EC	—	Garcia et al. (2016)
	*miR-208a* secreted from CM-Exo induced CF proliferation and differentiation to myofibroblasts	Inhibition of *miR-208a* attenuated cardiac fibrosis and improved cardiac function in post-MI rats	Yang et al. (2018)

## Applications of Stem Cell Exosomes in Cardiovascular Disease Treatment

After decades of research into stem cell therapy, research into exosomes has started to gain attention as a tool to counteract CVDs (Sahoo and Losordo, 2014; Wang et al., 2020). These natural nanocarriers are exploited as sources of biomarkers present in biofluids and as therapeutic drug delivery vehicles (Figures 1B–D).

## Exosomal Biomarkers in Cardiovascular Diseases

Biomarkers are molecules found in various biofluids that reflect the pathophysiological state of an individual, indicate the risk of future adverse events and predict response to treatment (Xue et al., 2020; Rezaie et al., 2021). In the past, several biomarkers have been identified for CVDs, of which cardiac Troponin T and I are considered the most reliable (Babuin and Jaffe 2005). However, in a large cohort study, their concentrations were found to vary between genders and in different age groups (Welsh et al., 2018). Hence, stable biomarkers are sought for CVD diagnosis. Several molecular studies reported alteration of exosomal protein cargos in various CVDs. For example, a proteome study of microvesicles of MI patients revealed modulated expression of 252 proteins, of which six proteins were key regulators of MI progression (Cheow et al., 2016). Compared to healthy subjects, blood exosomes of atherosclerosis patients were differentially enriched in proteasomal proteins such as PSMA6, PSMA7, and Annexin 2A (Jiang et al., 2016). Similarly, exosomal miRNAs (Exo-miR) are being extensively investigated for their diagnostic potential, especially cardiac miRs, which are known to be upregulated during CVDs (Colpaert and Calore 2019). The half-life of these miRs may vary in the patient’s serum/plasma, affecting their detection. However, crucial cardiac miRs released post-injury are reportedly carried safely by exosomes. Compared to circulating miRs released from blood cells and to some extent by dying cells (Zhou et al., 2018), the Exo-miRs are more stable and resistant to harsh environments because of their surrounding lipid envelope, hence are extremely promising as next-generation biomarkers.

Several preclinical studies indicate that Exo-miRs have superior diagnostic and prognostic value over unenveloped miRs (Gallo et al., 2012). In a retrospective multicenter study on HF patients, p53-responsive miRNAs, *miR-192*, *miR-194*, and *miR-34a* were found to be upregulated in exosomal fractions isolated from HF patients compared to controls. The authors advice further investigations with increased sample size and in other cohorts to confirm the present findings and future clinical applications of the Exo-miRs as predictive indicators of HF (Matsumoto et al., 2013). The serum exosomes of patients with ST-Elevation Myocardial Infarction (STEMI) showed increased levels of lncRNA, *NEAT1* and *miR-204* compared to controls. However, the specificity and sensitivity being low, serum exosomal *NEAT1*, *miR-204*, and MMP9 are considered useful only for auxiliary diagnosis of acute STEMI (Chen et al., 2020). In AMI mice, increased circulating levels of myocardial *miR-1*, *208b*, and *499* were predominantly present in circulating exosomes and *miR-133* in the non-exosomal component. Notably, these results were reproduced in humans as acute STEMI significantly increased circulating exosomal myo-miRs. However its clinical translation is still pending (Cheng et al., 2019)**.** Recently, various serum Exo-miRs; *miR-103a, 107, 320d, 486* and *let7b* were highly upregulated and acted as fair predictors of Atrial fibrillation progression (Mun et al., 2019). Prediction of HF, which is the terminal point of all heart ailments, still remains elusive, especially in the presence of other co-morbidities, e.g., obesity, diabetes, etc., which also affect the levels of Exo-miR (Patil et al., 2019). Plasma *Exo-miR425* and *Exo-miR744* are negative regulators of fibrosis and predictors of fibrosis and HF (Wang L. et al., 2018). Similarly, serum exosomal *miR-92b* is a potent marker of HF resulting from dilated cardiomyopathy, which still remains tough to diagnose due to the lack of suitable molecular diagnostics (Wu et al., 2018). To summarize, the elevated concentrations of various miRNAs in exosomal fractions have been increasingly observed during CVDs as shown in Figure 1B. However majority of investigators have suggested the need for follow-up studies in larger cohorts to confirm the clinical utility of the potential biomarkers studied by various research groups. Ongoing clinical trials include NCT03034265, where exosomal biomarkers are investigated for monitoring hypertension (ClinicalTrails.gov 2017); NCT04127591, where miRNA profiles of plasma exosomes are analyzed in MI patients (ClinicalTrails.gov 2019); the outcome of such studies is much awaited. Thus, despite accelerating research in the area, more detailed clinical investigations are needed along with multi-center studies pertaining to larger patient populations especially focused on exosomes in biofluids for faster bench to bedside conversion.

## Therapeutic Delivery

Targeted delivery of therapeutic exosomes is crucial for the effective treatment of CVDs. Earlier, synthetic carriers such as liposomes or polymeric nanoparticles have been widely used for drug delivery (Matoba et al., 2017). Recently, exosomes have been used to carry therapeutic molecules to the target tissue and have been found safe in preclinical studies (Gallet et al., 2017; Hirai et al., 2020). Although exosomes are endowed with several unique properties suitable for an ideal delivery system (Yang et al., 2015; Bellin et al., 2019), they have potential limitations as well. In this context, suboptimal biodistribution and low retention at the site of injury, along with a shorter half-life, are critical bottlenecks. Additionally, due to their uptake by the resident macrophages inside the spleen, lungs, and liver, they lead to their accumulation followed by clearance, thus forbidding their reach to the target site (Saunderson et al., 2014; Qiu et al., 2019).

To overcome these issues, researchers have implemented various nanotechnology and genetic engineering-based approaches to enhance the biodistribution, organ-specific targeting, and efficacy of exosomes. The biodistribution of endogenous exosomes was manipulated using magnetic nanoparticles that led to the accumulation of exosomes expressing CD63 in the injured CM (Liu et al., 2020). A recent study demonstrated that delivery of exosomes loaded with Clatherin-specific siRNA initially blocked their uptake by macrophages. Subsequent injection of exosomes loaded with therapeutic molecules bypassed the liver or spleen and reached the myocardium, as shown in the doxorubicin-induced cardiotoxicity model (Wan et al., 2020). To ensure cardiac homing, transmembrane proteins of exosomes were restructured by conjugating them with “cardiac homing peptide (CHP)” i.e., CSTSMLKAC. Upon their intravenous administration, exosomal retention in the infarcted myocardium was significantly improved (Vandergriff et al., 2018). However, the mechanism of CHP interaction with myocardium is still unknown. Likewise, MSC-Exo fused with “ischemic myocardium targeted peptide” enhanced the specificity and efficiency of their targeting (Wang X. et al., 2016). Intramyocardial administration of exosomes is encouraged instead of systemic administration, as the later led to an accumulation of exosomes in the liver (Bala et al., 2015). Interestingly, CPCs were genetically modified to overexpress CXCR4 for homing of exosomes to the injured myocardium. Intravenous administration of CPC^CXCR4^- Exo resulted in efficient CM survival *in vitro*, uptake by CM *ex vivo* and improved LV function in I/R rats (Ciullo et al., 2019). Since pathological remodeling of heart is predominantly contributed by loss of CM, therefore, engineering of the endogenous cardiac exosomes can enhance their retention in the heart. Recently, CDC-Exo was engineered to express Lamp2b and fused with cardiomyocyte specific binding peptide (CMP). As a result, the uptake of CMP tagged exosomes into CM was enhanced, following decreased apoptosis and increased retention in the heart in comparison to the non-targeted ones (Mentkowski and Lang 2019). Thus, engineering exosomes for organ-specific or cell-specific delivery offers new hope for the development of targeted therapeutics.

Several strategies are used to modify the therapeutic properties of exosomes. Stem cell exosomes can be loaded via electroporation, sonication, co-incubation or stimulation with cardioprotective agents, resulting in reparative exosomes that are taken up during biogenesis (Luan et al., 2017). Recently, Huang et al. have shown that atorvastatin pre-treated MSC-Exo showed superior cardioprotective benefits *in vitro* than the untreated ones and improved systolic function of the infarcted heart in AMI rats (Huang P. et al., 2020). The authors have identified lncRNA H19 as the key mediator of pro-angiogenesis resulting from atorvastatin treatment.

Functional regeneration of the myocardium largely relies on the local concentration of exosomes. Low retention due to rapid clearance and short circulation half-life (Ibrahim and Marbán 2016) diminishes their systemic biodistribution. Although a comparative study on exosome delivery showed greater myocardial retention *via* intramyocardial injection than intracoronary (Gallet et al., 2017), continuous administration of exosomes by such invasive procedures is clinically challenging. Tissue engineering approaches are being exploited to enhance the retention time by facilitating the controlled release of exosomes from the target region (Vader et al., 2016). In this regard, biomaterials such as hydrogels have emerged as a preferred choice as they are biodegradable, biocompatible, and able to slowly release the encapsulated exosomes at the injected site (Han et al., 2020). Endothelial progenitor cell exosomes encapsulated in hydrogel showed sustained release of exosomes for an extended period when injected into the ischemic myocardium (Chen et al., 2018). Additionally, cardiac patches have been used to deliver stem cells or therapeutics for cardiac retention (Seif-Naraghi et al., 2013). Interestingly, an acellular artificial cardiac patch was developed using a decellularized porcine extracellular matrix scaffold and synthetic cardiac stromal cells that promoted cardiac recovery in a rat AMI model (Huang K. et al., 2020). Furthermore, direct injection of exosome encapsulated hydrogel into the pericardial cavity forms a cardiac patch *in situ,* proving to be an effective and minimally invasive way for therapeutic delivery (Zhu et al., 2021). Thus, engineering exosomes in hydrogel offers a minimally invasive method for myocardial recovery (Figure 1C).

Though natural exosomes serve as an excellent system for delivery of therapeutic molecules, their insufficient number, heterogeneity, and scalability are major setbacks for clinical translation. Therefore, nanomedicine approaches are implemented to synthesize exosome mimetics tailored with the advantages of natural exosomes and synthetic components (Armstrong and Stevens, 2018; Villarreal-Leal et al., 2021). Engineered exosomes can be modified to contain a “Do not eat me” signal to increase the circulation time and avoid clearance by the immune system (Sun et al., 2020). Biodistribution of biomimetic nanoparticles was improved by coating nanoparticles with the RBC membrane, making them non-immunogenic, increasing circulation time and avoiding clearance by liver or immune cells (Liang et al., 2018). For controlled release of cargo at the target site, enzyme responsive strategies were developed to deliver therapeutic molecules to the infarcted heart using matrix metalloproteinase (MMP) enzyme in a minimally invasive way. When injected intravenously, biomimetic nanoparticles reached the infarcted area and released the therapeutic content based on environmental levels of MMP (Nguyen et al., 2015). Platelet inspired nano cells (PINC) where a platelet membrane with modified prostaglandin E2 exerted specific homing ability and incorporated the cardiac stromal cell secreted factors that specifically targeted the heart with ischemic injury (Su et al., 2019). Taken together, modifying the innate exosome properties *via* genetic engineering or nanotechnology may overcome their limitations and offer new hope for effective CVD treatment (Figure 1D).

## Conclusion and Future Perspectives

Stem cell exosomes seem promising as candidates for mending the broken heart. Over a decade of ongoing research on stem cells has exhibited improvements in the damaged heart, yet stem cell-derived acellular nanocarriers stand superior in terms of stability, bioavailability, and storage. Exosomes exert pleiotropic effects on the damaged myocardium by improving neoangiogenesis, cardiac conductance, autophagy, and suppressing inflammation and apoptosis, leading to functional improvements. While such studies were performed in smaller animal groups, more studies are desired in larger groups to delineate their true efficacy. The major challenge facing exosome research is the large-scale production of clinical grade exosomes, which is hindered by a lack of standard isolation techniques, heterogeneity, and low exosome yield. Interestingly, a recent attempt to develop large-scale production of GMP-grade exosomes from CPC-Exo has been appealing (Andriolo et al., 2018). More such studies are desired in this area, for an optimized and cost-effective protocol for sufficient production of exosomes, which may possibly meet the clinical needs. Exosomes in circulation holds great diagnostic potential as early biomarkers because of their increasingly proven abundance and stability in pathological milieu. As exosomes tend to interact with cells in different ways, either *via* receptor mediated interaction or internalization or *via* fusion with the cytoplasmic membrane, the therapeutic efficacy of drug-loaded exosomes is largely dependent on the mode of interaction. Consequently, understanding the molecular mechanisms will eventually improve selective drug targeting, and prevent off-target side effects. Besides tailoring exosomes with desirable properties, using novel engineering approaches and biomimetics platforms may offer potential solutions to circumvent the challenges associated with natural exosomes like unwanted side effects, biodistribution and clearance. As the exosome-mimetics field is advancing rapidly with innovative strategies, exosome-based therapeutics will not remain far from meeting clinical needs. However, more research is needed in this area to unleash the prospects of exosomes in CVD treatment.

## Glossary

ALLSTAR: ALLogenic Heart STem Cells to Achieve Myocardial Regeneration
AMI: acute myocardial infarction
ANF: atrial natriuretic factor
BM: bone marrow
BM-Exo: bone marrow exosome
BNP: brain natriuretic peptide
CADUCEUS: Cardiosphere-Derived Autologous Stem Cells to Reverse Ventricular Dysfunction
CDC: cardiosphere-derived cell
CDC-Exo: cardiosphere-derived cell exosome
CF: cardiac fibroblast
CM: cardiomyocyte
CM-Exo: cardiomyocyte exosome
CMP: cardiomyocyte binding protein
CPC: cardiac progenitor cell
CPC-Exo: cardiac progenitor cell exosome
DCM: dilated cardiomyopathy
ECs: endothelial cells
ECM: extracellular matrix protein
ESCs: embryonic stem cells
ESC-Exo: embryonic stem cell-derived exosome
ESC-Pg-Exo: embryonic stem cell-derived cardiovascular progenitor exosome
Exo-miR: exosome-derived miRNA
FS: fractional shortening
HF: heart failure
HSP: heat-shock proteins
I/R: ischemia/reperfusion
ICM: ischemic cardiomyopathy
iCM: induced pluripotent stem cell-derived cardiomyocyte
iCM-Exo: induced pluripotent stem cell-derived cardiomyocyte exosome
iPSC: induced pluripotent stem cell
iPSC-CC: induced pluripotent stem cell-derived cardiac cell
iPSC-Exo: induced pluripotent stem cell exosome
LVEF: left ventricular ejection fraction
LVESV/LVEDV: left ventricular end systolic volume/ left ventricular end diastolic volume
MI: myocardial infarction
MIR: myocardial ischemia–reperfusion
miRNA/miR: micro-RNA
MMP: matrix metalloproteinase
MSCs: mesenchymal stem cells
MSC-Exo: mesenchymal stem cell exosome
NVRM: neonatal ventricular myocyte
PAPP-A: pregnancy-associated plasma protein-A
STEMI: ST-elevation myocardial infarction

## References

[B1] Adamiak M. Cheng G. Bobis-Wozowicz S. Zhao L. Kedracka-Krok S. Samanta A. (2018). Induced Pluripotent Stem Cell (iPSC)-Derived Extracellular Vesicles Are Safer and More Effective for Cardiac Repair Than iPSCs. Circ. Res. 122 (2), 296–309. 10.1161/circresaha.117.311769 29118058 PMC5775034

[B2] Andriolo G. Provasi E. Lo Cicero V. Brambilla A. Soncin S. Torre T. (2018). Exosomes from Human Cardiac Progenitor Cells for Therapeutic Applications: Development of a GMP-Grade Manufacturing Method. Front. Physiol. 9, 1169. 10.3389/fphys.2018.01169 30197601 PMC6117231

[B3] Armstrong J. P. K. Stevens M. M. (2018). Strategic Design of Extracellular Vesicle Drug Delivery Systems. Adv. Drug Deliv. Rev. 130, 12–16. 10.1016/j.addr.2018.06.017 29959959 PMC6606438

[B4] Babuin L. Jaffe A. S. (2005). Troponin: the Biomarker of Choice for the Detection of Cardiac Injury. Cmaj 173 (10), 1191–1202. 10.1503/cmaj/05129110.1503/cmaj.050141 16275971 PMC1277047

[B5] Bala S. Csak T. Momen-Heravi F. Lippai D. Kodys K. Catalano D. (2015). Biodistribution and Function of Extracellular miRNA-155 in Mice. Sci. Rep. 5, 10721. 10.1038/srep10721 26024046 PMC4448655

[B6] Balbi C. Vassalli G. (2020). Exosomes: Beyond Stem Cells for Cardiac Protection and Repair. Stem Cells 38 (11), 1387–1399. 10.1002/stem.3261 32761640

[B7] Banerjee M. N. Bolli R. Hare J. M. (2018). Clinical Studies of Cell Therapy in Cardiovascular Medicine. Circ. Res. 123 (2), 266–287. 10.1161/circresaha.118.311217 29976692 PMC8742222

[B8] Barile L. Cervio E. Lionetti V. Milano G. Ciullo A. Biemmi V. (2018). Cardioprotection by Cardiac Progenitor Cell-Secreted Exosomes: Role of Pregnancy-Associated Plasma Protein-A. Cardiovasc Res. 114 (7), 992–1005. 10.1093/cvr/cvy055 29518183

[B9] Barile L. Vassalli G. (2017). Exosomes: Therapy Delivery Tools and Biomarkers of Diseases. Pharmacol. Ther. 174, 63–78. 10.1016/j.pharmthera.2017.02.020 28202367

[B10] Bellin G. Gardin C. Ferroni L. Chachques J. Rogante M. Mitrečić D. (2019). Exosome in Cardiovascular Diseases: A Complex World Full of Hope. Cells 8 (2), 166. 10.3390/cells8020166 PMC6406975 30781555

[B11] Capriotti T. Micari M. (2019). Chronic Heart Failure Treatment with the Left Ventricular Assist Device. Home Healthc. Now. 37 (4), 190–197. 10.1097/nhh.0000000000000777 31274581

[B12] Charles C. J. Li R. R. Yeung T. Mazlan S. M. I. Lai R. C. de Kleijn D. P. V. (2020). Systemic Mesenchymal Stem Cell-Derived Exosomes Reduce Myocardial Infarct Size: Characterization with MRI in a Porcine Model. Front. Cardiovasc. Med. 7, 601990. 10.3389/fcvm.2020.601990 33304934 PMC7701257

[B13] Chen C. W. Wang L. L. Zaman S. Gordon J. Arisi M. F. Venkataraman C. M. (2018). Sustained Release of Endothelial Progenitor Cell-Derived Extracellular Vesicles from Shear-Thinning Hydrogels Improves Angiogenesis and Promotes Function after Myocardial Infarction. Cardiovasc Res. 114 (7), 1029–1040. 10.1093/cvr/cvy067 29566124 PMC5967544

[B14] Chen L. Wang Y. Pan Y. Zhang L. Shen C. Qin G. (2013). Cardiac Progenitor-Derived Exosomes Protect Ischemic Myocardium from Acute Ischemia/reperfusion Injury. Biochem. Biophysical Res. Commun. 431 (3), 566–571. 10.1016/j.bbrc.2013.01.015 PMC3732190 23318173

[B15] Chen Z. Yan Y. Wu J. Qi C. Liu J. Wang J. (2020). Expression Level and Diagnostic Value of Exosomal NEAT1 /miR ‐204/MMP ‐9 in Acute ST ‐segment Elevation Myocardial Infarction. IUBMB Life 72 (11), 2499–2507. 10.1002/iub.2376 32916037

[B16] Cheng M. Yang J. Zhao X. Zhang E. Zeng Q. Yu Y. (2019). Circulating Myocardial microRNAs from Infarcted Hearts Are Carried in Exosomes and Mobilise Bone Marrow Progenitor Cells. Nat. Commun. 10 (1), 959. 10.1038/s41467-019-08895-7 30814518 PMC6393447

[B17] Cheow E. S. H. Cheng W. C. Lee C. N. de Kleijn D. Sorokin V. Sze S. K. (2016). Plasma-derived Extracellular Vesicles Contain Predictive Biomarkers and Potential Therapeutic Targets for Myocardial Ischemic (MI) Injury. Mol. Cell. Proteomics 15 (8), 2628–2640. 10.1074/mcp.M115.055731 27234505 PMC4974341

[B18] Ciullo A. Biemmi V. Milano G. Bolis S. Cervio E. Fertig E. T. (2019). Exosomal Expression of CXCR4 Targets Cardioprotective Vesicles to Myocardial Infarction and Improves Outcome after Systemic Administration. Ijms 20 (3), 468. 10.3390/ijms20030468 PMC6386845 30678240

[B19] ClinicalTrails.gov (2019). Differential Expression and Analysis of Peripheral Plasma Exosome miRNA in Patients with Myocardial Infarction. Available at: https://clinicaltrials.gov/ct2/show/NCT04127591 (Retrieved October 15, 2019).

[B20] ClinicalTrails.gov (2017). New Biomarkers and Difficult-To-Treat Hypertension. Available at: https://clinicaltrials.gov/ct2/show/NCT03034265 (Retrieved December 20, 2017).

[B21] Colpaert R. M. W. Calore M. (2019). MicroRNAs in Cardiac Diseases. Cells 8 (7), 737. 10.3390/cells8070737 PMC6678080 31323768

[B22] Cui X. He Z. Liang Z. Chen Z. Wang H. Zhang J. (2017). Exosomes from Adipose-Derived Mesenchymal Stem Cells Protect the Myocardium against Ischemia/Reperfusion Injury Through Wnt/β-Catenin Signaling Pathway. J. Cardiovasc Pharmacol. 70 (4), 225–231. 10.1097/fjc.0000000000000507 28582278 PMC5642342

[B23] El Harane N. Kervadec A. Bellamy V. Pidial L. Neametalla H. J. Perier M. C. (2018). Acellular Therapeutic Approach for Heart Failure: *In Vitro* Production of Extracellular Vesicles from Human Cardiovascular Progenitors. Eur. Heart J. 39 (20), 1835–1847. 10.1093/eurheartj/ehy012 29420830 PMC6251654

[B24] Gallet R. Dawkins J. Valle J. Simsolo E. de Couto G. Middleton R. (2017). Exosomes Secreted by Cardiosphere-Derived Cells Reduce Scarring, Attenuate Adverse Remodelling, and Improve Function in Acute and Chronic Porcine Myocardial Infarction. Eur. Heart J. 38 (3), ehw240–211. 10.1093/eurheartj/ehw240 PMC5837390 28158410

[B25] Gallo A. Tandon M. Alevizos I. Illei G. G. (2012). The Majority of microRNAs Detectable in Serum and Saliva Is Concentrated in Exosomes. PLoS One 7 (3), e30679. 10.1371/journal.pone.0030679 22427800 PMC3302865

[B26] Gao L. Wang L. Wei Y. Krishnamurthy P. Walcott G. P. Menasché P. (2020). Exosomes Secreted by hiPSC-Derived Cardiac Cells Improve Recovery from Myocardial Infarction in Swine. Sci. Transl. Med. 12 (561), eaay1318. 10.1126/scitranslmed.aay1318 32938792

[B27] Garcia N. A. Moncayo-Arlandi J. Sepulveda P. Diez-Juan A. (2016). Cardiomyocyte Exosomes Regulate Glycolytic Flux in Endothelium by Direct Transfer of GLUT Transporters and Glycolytic Enzymes. Cardiovasc Res. 109 (3), 397–408. 10.1093/cvr/cvv260 26609058

[B28] Gnecchi M. Zhang Z. Ni A. Dzau V. J. (2008). Paracrine Mechanisms in Adult Stem Cell Signaling and Therapy. Circulation Res. 103 (11), 1204–1219. 10.1161/circresaha.108.176826 19028920 PMC2667788

[B29] Gray W. D. French K. M. Ghosh-Choudhary S. Maxwell J. T. Brown M. E. Platt M. O. (2015). Identification of Therapeutic Covariant microRNA Clusters in Hypoxia-Treated Cardiac Progenitor Cell Exosomes Using Systems Biology. Circ. Res. 116 (2), 255–263. 10.1161/circresaha.116.304360 25344555 PMC4338016

[B30] Gupta S. Knowlton A. A. (2007). HSP60 Trafficking in Adult Cardiac Myocytes: Role of the Exosomal Pathway. Am. J. Physiology-Heart Circulatory Physiology 292 (6), H3052–H3056. 10.1152/ajpheart.01355.2006 17307989

[B31] Han C. Zhang Z. Sun J. Li K. Li Y. Ren C. (2020). Self-Assembling Peptide-Based Hydrogels in Angiogenesis. Ijn 15, 10257–10269. 10.2147/ijn.S277046 33364757 PMC7751603

[B32] Hare J. M. DiFede D. L. Rieger A. C. Florea V. Landin A. M. El-Khorazaty J. (2017). Randomized Comparison of Allogeneic versus Autologous Mesenchymal Stem Cells for Nonischemic Dilated Cardiomyopathy. J. Am. Coll. Cardiol. 69 (5), 526–537. 10.1016/j.jacc.2016.11.009 27856208 PMC5291766

[B33] Hassanpour M. Rezabakhsh A. Rezaie J. Nouri M. Rahbarghazi R. (2020). Exosomal Cargos Modulate Autophagy in Recipient Cells via Different Signaling Pathways. Cell Biosci. 10, 92. 10.1186/s13578-020-00455-7 32765827 PMC7395405

[B34] Henning R. J. (2021). Cardiovascular Exosomes and MicroRNAs in Cardiovascular Physiology and Pathophysiology. J. Cardiovasc. Trans. Res. 14 (2), 195–212. 10.1007/s12265-020-10040-5 32588374

[B35] Hirai K. Ousaka D. Fukushima Y. Kondo M. Eitoku T. Shigemitsu Y. (2020). Cardiosphere-derived Exosomal microRNAs for Myocardial Repair in Pediatric Dilated Cardiomyopathy. Sci. Transl. Med. 12 (573), eabb3336. 10.1126/scitranslmed.abb3336 33298561

[B36] Huang K. Ozpinar E. W. Su T. Tang J. Shen D. Qiao L. (2020). An Off-The-Shelf Artificial Cardiac Patch Improves Cardiac Repair after Myocardial Infarction in Rats and Pigs. Sci. Transl. Med. 12 (538). 10.1126/scitranslmed.aat9683 PMC7293901 32269164

[B37] Huang P. Wang L. Li Q. Tian X. Xu J. Xu J. (2020). Atorvastatin Enhances the Therapeutic Efficacy of Mesenchymal Stem Cells-Derived Exosomes in Acute Myocardial Infarction via Up-Regulating Long Non-coding RNA H19. Cardiovasc Res. 116 (2), 353–367. 10.1093/cvr/cvz139 31119268 PMC8204482

[B38] Ibrahim A. G.-E. Cheng K. Marbán E. (2014). Exosomes as Critical Agents of Cardiac Regeneration Triggered by Cell Therapy. Stem Cell Rep. 2 (5), 606–619. 10.1016/j.stemcr.2014.04.006 PMC4050492 24936449

[B39] Ibrahim A. Marbán E. (2016). Exosomes: Fundamental Biology and Roles in Cardiovascular Physiology. Annu. Rev. Physiol. 78, 67–83. 10.1146/annurev-physiol-021115-104929 26667071 PMC5425157

[B40] Jiang M. Quan J. Zhang H. Ding Q. Xiang M. Meng D. (2016). Proteomic Identification of Proteins in Exosomes of Patients with Atherosclerosis. Chin. J. Pathophysiol. 24, 1525–1526.

[B41] Jung J.-H. Fu X. Yang P. C. (2017). Exosomes Generated from iPSC-Derivatives. Circ. Res. 120 (2), 407–417. 10.1161/circresaha.116.309307 28104773 PMC5260934

[B42] Karantalis V. Hare J. M. (2015). Use of Mesenchymal Stem Cells for Therapy of Cardiac Disease. Circ. Res. 116 (8), 1413–1430. 10.1161/circresaha.116.303614 25858066 PMC4429294

[B43] Kervadec A. Bellamy V. El Harane N. Arakélian L. Vanneaux V. Cacciapuoti I. (2016). Cardiovascular Progenitor-Derived Extracellular Vesicles Recapitulate the Beneficial Effects of Their Parent Cells in the Treatment of Chronic Heart Failure. J. Heart Lung Transplant. 35 (6), 795–807. 10.1016/j.healun.2016.01.013 27041495

[B44] Khaksar M. Sayyari M. Rezaie J. Pouyafar A. Montazersaheb S. Rahbarghazi R. (2018). High Glucose Condition Limited the Angiogenic/cardiogenic Capacity of Murine Cardiac Progenitor Cells in *In Vitro* and *In Vivo* Milieu. Cell Biochem. Funct. 36 (7), 346–356. 10.1002/cbf.3354 30051492

[B45] Khan M. Nickoloff E. Abramova T. Johnson J. Verma S. K. Krishnamurthy P. (2015). Embryonic Stem Cell-Derived Exosomes Promote Endogenous Repair Mechanisms and Enhance Cardiac Function Following Myocardial Infarction. Circ. Res. 117 (1), 52–64. 10.1161/circresaha.117.305990 25904597 PMC4482130

[B46] Kishore R. Khan M. (2017). Cardiac Cell-Derived Exosomes: Changing Face of Regenerative Biology. Eur. Heart J. 38 (3), ehw324–215. 10.1093/eurheartj/ehw324 PMC5837427 28158461

[B47] Kishore R. Khan M. (2016). More Than Tiny Sacks. Circ. Res. 118 (2), 330–343. 10.1161/circresaha.115.307654 26838317 PMC4743531

[B48] Kowal J. Tkach M. Théry C. (2014). Biogenesis and Secretion of Exosomes. Curr. Opin. Cell Biol. 29, 116–125. 10.1016/j.ceb.2014.05.004 24959705

[B49] Kurtzwald-Josefson E. Zeevi-Levin N. Rubchevsky V. Bechar Erdman N. Schwartz Rohaker O. Nahum O. (2020). Cardiac Fibroblast-Induced Pluripotent Stem Cell-Derived Exosomes as a Potential Therapeutic Mean for Heart Failure. Ijms 21 (19), 7215. 10.3390/ijms21197215 PMC7582575 33003641

[B50] Lalit P. A. Hei D. J. Raval A. N. Kamp T. J. (2014). Induced Pluripotent Stem Cells for Post-Myocardial Infarction Repair. Circ. Res. 114 (8), 1328–1345. 10.1161/circresaha.114.300556 24723658 PMC4016859

[B51] Lee W. H. Chen W.-Y. Shao N.-Y. Xiao D. Qin X. Baker N. (2017). Comparison of Non-Coding RNAs in Exosomes and Functional Efficacy of Human Embryonic Stem Cell- versus Induced Pluripotent Stem Cell-Derived Cardiomyocytes. Stem Cells 35 (10), 2138–2149. 10.1002/stem.2669 28710827 PMC5918285

[B52] Li P. Kaslan M. Lee S. H. Yao J. Gao Z. (2017). Progress in Exosome Isolation Techniques. Theranostics 7 (3), 789–804. 10.7150/thno.18133 28255367 PMC5327650

[B53] Li T.-S. Cheng K. Malliaras K. Smith R. R. Zhang Y. Sun B. (2012). Direct Comparison of Different Stem Cell Types and Subpopulations Reveals Superior Paracrine Potency and Myocardial Repair Efficacy with Cardiosphere-Derived Cells. J. Am. Coll. Cardiol. 59 (10), 942–953. 10.1016/j.jacc.2011.11.029 22381431 PMC3292778

[B54] Li X. Yang Z. Nie W. Jiang J. Li S. Li Z. (2019). Exosomes Derived from Cardiac Progenitor Cells Attenuate CVB3-Induced Apoptosis via Abrogating the Proliferation of CVB3 and Modulating the mTOR Signaling Pathways. Cell Death Dis. 10 (10), 691. 10.1038/s41419-019-1910-9 31534118 PMC6751166

[B55] Liang H. Huang K. Su T. Li Z. Hu S. Dinh P.-U. (2018). Mesenchymal Stem Cell/Red Blood Cell-Inspired Nanoparticle Therapy in Mice with Carbon Tetrachloride-Induced Acute Liver Failure. ACS Nano 12 (7), 6536–6544. 10.1021/acsnano.8b00553 29943967 PMC6373867

[B56] Liu L. Jin X. Hu C.-F. Li R. Zhou Z. e. Shen C.-X. (2017). Exosomes Derived from Mesenchymal Stem Cells Rescue Myocardial Ischaemia/Reperfusion Injury by Inducing Cardiomyocyte Autophagy via AMPK and Akt Pathways. Cell Physiol. Biochem. 43 (1), 52–68. 10.1159/000480317 28848091

[B57] Liu S. Chen X. Bao L. Liu T. Yuan P. Yang X. (2020). Treatment of Infarcted Heart Tissue via the Capture and Local Delivery of Circulating Exosomes through Antibody-Conjugated Magnetic Nanoparticles. Nat. Biomed. Eng. 4 (11), 1063–1075. 10.1038/s41551-020-00637-1 33159193

[B58] Luan X. Sansanaphongpricha K. Myers I. Chen H. Yuan H. Sun D. (2017). Engineering Exosomes as Refined Biological Nanoplatforms for Drug Delivery. Acta Pharmacol. Sin. 38 (6), 754–763. 10.1038/aps.2017.12 28392567 PMC5520184

[B59] Makkar R. R. Kereiakes D. J. Aguirre F. Kowalchuk G. Chakravarty T. Malliaras K. (2020). Intracoronary ALLogeneic Heart STem Cells to Achieve Myocardial Regeneration (ALLSTAR): a Randomized, Placebo-Controlled, Double-Blinded Trial. Eur. Heart J. 41 (36), 3451–3458. 10.1093/eurheartj/ehaa541 32749459

[B60] Makkar R. R. Smith R. R. Cheng K. Malliaras K. Thomson L. E. Berman D. (2012). Intracoronary Cardiosphere-Derived Cells for Heart Regeneration after Myocardial Infarction (CADUCEUS): a Prospective, Randomised Phase 1 Trial. Lancet 379 (9819), 895–904. 10.1016/s0140-6736(12)60195-0 22336189 PMC4326004

[B61] Marbán E. (2018). The Secret Life of Exosomes: What Bees Can Teach Us about Next-Generation Therapeutics. J. Am. Coll. Cardiol. 71 (2), 193–200. 10.1016/j.jacc.2017.11.013 29325643 PMC5769161

[B62] Matoba T. Koga J.-i. Nakano K. Egashira K. Tsutsui H. (2017). Nanoparticle-mediated Drug Delivery System for Atherosclerotic Cardiovascular Disease. J. Cardiol. 70 (3), 206–211. 10.1016/j.jjcc.2017.03.005 28416142

[B63] Matsumoto S. Sakata Y. Suna S. Nakatani D. Usami M. Hara M. (2013). Circulating P53-Responsive microRNAs Are Predictive Indicators of Heart Failure after Acute Myocardial Infarction. Circ. Res. 113 (3), 322–326. 10.1161/circresaha.113.301209 23743335

[B64] Menasché P. (2018). Cell Therapy Trials for Heart Regeneration - Lessons Learned and Future Directions. Nat. Rev. Cardiol. 15 (11), 659–671. 10.1038/s41569-018-0013-0 29743563

[B65] Mentkowski K. I. Lang J. K. (2019). Exosomes Engineered to Express a Cardiomyocyte Binding Peptide Demonstrate Improved Cardiac Retention *In Vivo* . Sci. Rep. 9 (1), 10041. 10.1038/s41598-019-46407-1 31296886 PMC6624248

[B66] Messina E. De Angelis L. Frati G. Morrone S. Chimenti S. Fiordaliso F. (2004). Isolation and Expansion of Adult Cardiac Stem Cells from Human and Murine Heart. Circulation Res. 95 (9), 911–921. 10.1161/01.Res.0000147315.71699.51 15472116

[B67] Milano G. Biemmi V. Lazzarini E. Balbi C. Ciullo A. Bolis S. (2020). Intravenous Administration of Cardiac Progenitor Cell-Derived Exosomes Protects against Doxorubicin/trastuzumab-Induced Cardiac Toxicity. Cardiovasc Res. 116 (2), 383–392. 10.1093/cvr/cvz108 31098627

[B68] Müller P. Beltrami A. Cesselli D. Pfeiffer P. Kazakov A. Böhm M. (2005). Myocardial Regeneration by Endogenous Adult Progenitor Cells. J. Mol. Cell. Cardiol. 39 (2), 377–387. 10.1016/j.yjmcc.2005.03.001 16038708

[B69] Müller P. Lemcke H. David R. (2018). Stem Cell Therapy in Heart Diseases - Cell Types, Mechanisms and Improvement Strategies. Cell Physiol. Biochem. 48 (6), 2607–2655. 10.1159/000492704 30121644

[B70] Mun D. Kim H. Kang J. Y. Park H. Park H. Lee S. H. (2019). Expression of miRNAs in Circulating Exosomes Derived from Patients with Persistent Atrial Fibrillation. FASEB J. 33 (5), 5979–5989. 10.1096/fj.201801758R 30753098

[B71] Murphy D. E. de Jong O. G. Brouwer M. Wood M. J. Lavieu G. Schiffelers R. M. (2019). Extracellular Vesicle-Based Therapeutics: Natural versus Engineered Targeting and Trafficking. Exp. Mol. Med. 51 (3), 1–12. 10.1038/s12276-019-0223-5 PMC6418170 30872574

[B72] Nazari-Shafti T. Z. Exarchos V. Biefer H. R. C. Cesarovic N. Meyborg H. Falk V. (2020). MicroRNA Mediated Cardioprotection - Is There a Path to Clinical Translation? Front. Bioeng. Biotechnol. 8, 149. 10.3389/fbioe.2020.00149 32266222 PMC7099408

[B73] Nguyen M. M. Carlini A. S. Chien M.-P. Sonnenberg S. Luo C. Braden R. L. (2015). Enzyme-Responsive Nanoparticles for Targeted Accumulation and Prolonged Retention in Heart Tissue after Myocardial Infarction. Adv. Mat. 27 (37), 5547–5552. 10.1002/adma.201502003 PMC4699559 26305446

[B74] Ong S.-G. Wu J. C. (2015). Exosomes as Potential Alternatives to Stem Cell Therapy in Mediating Cardiac Regeneration. Circ. Res. 117 (1), 7–9. 10.1161/circresaha.115.306593 26089361 PMC4546704

[B75] Patil M. Henderson J. Luong H. Annamalai D. Sreejit G. Krishnamurthy P. (2019). The Art of Intercellular Wireless Communications: Exosomes in Heart Disease and Therapy. Front. Cell Dev. Biol. 7, 315. 10.3389/fcell.2019.00315 31850349 PMC6902075

[B76] Perin E. C. Sanz-Ruiz R. Sánchez P. L. Lasso J. Pérez-Cano R. Alonso-Farto J. C. (2014). Adipose-derived Regenerative Cells in Patients with Ischemic Cardiomyopathy: The PRECISE Trial. Am. Heart J. 168 (1), 88–95.e2. 10.1016/j.ahj.2014.03.022 24952864

[B77] Pittenger M. F. Discher D. E. Péault B. M. Phinney D. G. Hare J. M. Caplan A. I. (2019). Mesenchymal Stem Cell Perspective: Cell Biology to Clinical Progress. NPJ Regen. Med. 4, 22. 10.1038/s41536-019-0083-6 31815001 PMC6889290

[B78] Qiu X. Li Z. Han X. Zhen L. Luo C. Liu M. (2019). Tumor-derived Nanovesicles Promote Lung Distribution of the Therapeutic Nanovector through Repression of Kupffer Cell-Mediated Phagocytosis. Theranostics 9 (9), 2618–2636. 10.7150/thno.32363 31131057 PMC6525995

[B79] Razeghian-Jahromi I. Matta A. G. Canitrot R. Zibaeenezhad M. J. Razmkhah M. Safari A. (2021). Surfing the Clinical Trials of Mesenchymal Stem Cell Therapy in Ischemic Cardiomyopathy. Stem Cell Res. Ther. 12 (1), 361. 10.1186/s13287-021-02443-1 34162424 PMC8220796

[B80] Rezaie J. Aslan C. Ahmadi M. Zolbanin N. M. Kashanchi F. Jafari R. (2021). The Versatile Role of Exosomes in Human Retroviral Infections: from Immunopathogenesis to Clinical Application. Cell Biosci. 11 (1), 19. 10.1186/s13578-021-00537-0 33451365 PMC7810184

[B81] Rezaie J. Rahbarghazi R. Pezeshki M. Mazhar M. Yekani F. Khaksar M. (2019). Cardioprotective Role of Extracellular Vesicles: A Highlight on Exosome Beneficial Effects in Cardiovascular Diseases. J. Cell. Physiology 234 (12), 21732–21745. 10.1002/jcp.28894 31140622

[B82] Ribeiro-Rodrigues T. M. Laundos T. L. Pereira-Carvalho R. Batista-Almeida D. Pereira R. Coelho-Santos V. (2017). Exosomes Secreted by Cardiomyocytes Subjected to Ischaemia Promote Cardiac Angiogenesis. Cardiovasc Res. 113 (11), 1338–1350. 10.1093/cvr/cvx118 28859292

[B83] Saha P. Sharma S. Korutla L. Datla S. R. Shoja-Taheri F. Mishra R. (2019). Circulating Exosomes Derived from Transplanted Progenitor Cells Aid the Functional Recovery of Ischemic Myocardium. Sci. Transl. Med. 11 (493), eaau1168. 10.1126/scitranslmed.aau1168 31118291 PMC6857931

[B84] Sahoo S. Adamiak M. Mathiyalagan P. Kenneweg F. Kafert-Kasting S. Thum T. (2021). Therapeutic and Diagnostic Translation of Extracellular Vesicles in Cardiovascular Diseases. Circulation 143 (14), 1426–1449. 10.1161/circulationaha.120.049254 33819075 PMC8021236

[B85] Sahoo S. Losordo D. W. (2014). Exosomes and Cardiac Repair after Myocardial Infarction. Circ. Res. 114 (2), 333–344. 10.1161/circresaha.114.300639 24436429

[B86] Santoso M. R. Ikeda G. Tada Y. Jung J. H. Vaskova E. Sierra R. G. (2020). Exosomes from Induced Pluripotent Stem Cell-Derived Cardiomyocytes Promote Autophagy for Myocardial Repair. Jaha 9 (6), e014345. 10.1161/jaha.119.014345 32131688 PMC7335524

[B87] Saunderson S. C. Dunn A. C. Crocker P. R. McLellan A. D. (2014). CD169 Mediates the Capture of Exosomes in Spleen and Lymph Node. Blood 123 (2), 208–216. 10.1182/blood-2013-03-489732 24255917 PMC3888287

[B88] Seif-Naraghi S. B. Singelyn J. M. Salvatore M. A. Osborn K. G. Wang J. J. Sampat U. (2013). Safety and Efficacy of an Injectable Extracellular Matrix Hydrogel for Treating Myocardial Infarction. Sci. Transl. Med. 5 (173), 173ra125. 10.1126/scitranslmed.3005503 PMC3848875 23427245

[B89] Shao L. Zhang Y. Lan B. Wang J. Zhang Z. Zhang L. (20172017). MiRNA-Sequence Indicates That Mesenchymal Stem Cells and Exosomes Have Similar Mechanism to Enhance Cardiac Repair. BioMed Res. Int. 2017, 9. 10.1155/2017/4150705 PMC5292186 28203568

[B90] Su T. Huang K. Ma H. Liang H. Dinh P. U. Chen J. (2019). Platelet‐Inspired Nanocells for Targeted Heart Repair after Ischemia/Reperfusion Injury. Adv. Funct. Mat. 29 (4), 1803567. 10.1002/adfm.201803567 PMC7111457 32256277

[B91] Sun J. Muz B. Alhallak K. Markovic M. Gurley S. Wang Z. (2020). Targeting CD47 as a Novel Immunotherapy for Multiple Myeloma. Cancers 12 (2), 305. 10.3390/cancers12020305 PMC7072283 32012878

[B92] Tompkins B. A. Rieger A. C. Florea V. Banerjee M. N. Natsumeda M. Nigh E. D. (2018). Comparison of Mesenchymal Stem Cell Efficacy in Ischemic versus Nonischemic Dilated Cardiomyopathy. Jaha 7 (14), e008460. 10.1161/jaha.117.008460 30005555 PMC6064862

[B93] Vader P. Mol E. A. Pasterkamp G. Schiffelers R. M. (2016). Extracellular Vesicles for Drug Delivery. Adv. Drug Deliv. Rev. 106 (Pt A), 148–156. 10.1016/j.addr.2016.02.006 26928656

[B94] Vandergriff A. Huang K. Shen D. Hu S. Hensley M. T. Caranasos T. G. (2018). Targeting Regenerative Exosomes to Myocardial Infarction Using Cardiac Homing Peptide. Theranostics 8 (7), 1869–1878. 10.7150/thno.20524 29556361 PMC5858505

[B95] Villarreal-Leal R. A. Cooke J. P. Corradetti B. (2021). Biomimetic and Immunomodulatory Therapeutics as an Alternative to Natural Exosomes for Vascular and Cardiac Applications. Nanomedicine Nanotechnol. Biol. Med. 35, 102385. 10.1016/j.nano.2021.102385 PMC8238887 33774130

[B96] Volarevic V. Markovic B. S. Gazdic M. Volarevic A. Jovicic N. Arsenijevic N. (2018). Ethical and Safety Issues of Stem Cell-Based Therapy. Int. J. Med. Sci. 15 (1), 36–45. 10.7150/ijms.21666 29333086 PMC5765738

[B97] Walravens A.-S. Smolgovsky S. Li L. Kelly L. Antes T. Peck K. (2021). Mechanistic and Therapeutic Distinctions between Cardiosphere-Derived Cell and Mesenchymal Stem Cell Extracellular Vesicle Non-coding RNA. Sci. Rep. 11 (1), 8666. 10.1038/s41598-021-87939-9 33883598 PMC8060398

[B98] Wan Z. Zhao L. Lu F. Gao X. Dong Y. Zhao Y. (2020). Mononuclear Phagocyte System Blockade Improves Therapeutic Exosome Delivery to the Myocardium. Theranostics 10 (1), 218–230. 10.7150/thno.38198 31903116 PMC6929612

[B99] Wang H. Xie Y. Salvador A. M. Zhang Z. Chen K. Li G. (2020). Exosomes: Multifaceted Messengers in Atherosclerosis. Curr. Atheroscler. Rep. 22 (10), 57. 10.1007/s11883-020-00871-7 32772195

[B100] Wang J. Chen J. Sen S. (2016). MicroRNA as Biomarkers and Diagnostics. J. Cell. Physiol. 231 (1), 25–30. 10.1002/jcp.25056 26031493 PMC8776330

[B101] Wang L. Liu J. Xu B. Liu Y.-L. Liu Z. (2018). Reduced Exosome miR-425 and miR-744 in the Plasma Represents the Progression of Fibrosis and Heart Failure. Kaohsiung J. Med. Sci. 34 (11), 626–633. 10.1016/j.kjms.2018.05.008 30392569 PMC11915641

[B102] Wang L. Zhang J. (2020). Exosomal lncRNA AK139128 Derived from Hypoxic Cardiomyocytes Promotes Apoptosis and Inhibits Cell Proliferation in Cardiac Fibroblasts. Ijn 15, 3363–3376. 10.2147/ijn.S240660 32494135 PMC7229807

[B103] Wang X. Chen Y. Zhao Z. Meng Q. Yu Y. Sun J. (2018). Engineered Exosomes with Ischemic Myocardium‐Targeting Peptide for Targeted Therapy in Myocardial Infarction. Jaha 7 (15), e008737. 10.1161/jaha.118.008737 30371236 PMC6201471

[B104] Wang X. Gu H. Huang W. Peng J. Li Y. Yang L. (2016). Hsp20-Mediated Activation of Exosome Biogenesis in Cardiomyocytes Improves Cardiac Function and Angiogenesis in Diabetic Mice. Diabetes 65 (10), 3111–3128. 10.2337/db15-1563 27284111 PMC5033265

[B105] Wang Y. Zhang L. Li Y. Chen L. Wang X. Guo W. (2015). Exosomes/microvesicles from Induced Pluripotent Stem Cells Deliver Cardioprotective miRNAs and Prevent Cardiomyocyte Apoptosis in the Ischemic Myocardium. Int. J. Cardiol. 192, 61–69. 10.1016/j.ijcard.2015.05.020 26000464 PMC4469495

[B106] Welsh P. Preiss D. Shah A. S. V. McAllister D. Briggs A. Boachie C. (2018). Comparison between High-Sensitivity Cardiac Troponin T and Cardiac Troponin I in a Large General Population Cohort. Clin. Chem. 64 (11), 1607–1616. 10.1373/clinchem.2018.292086 30126950 PMC6398571

[B107] WorldHealthOrganization (2021). Cardiovascular Diseases (CVDs). Available at: https://www.who.int/en/news-room/fact-sheets/detail/cardiovascular-diseases-(cvds) (Retrieved June 11, 2021).

[B108] Wu Q. Wang J. Tan W. L. W. Jiang Y. Wang S. Li Q. (2020). Extracellular Vesicles from Human Embryonic Stem Cell-Derived Cardiovascular Progenitor Cells Promote Cardiac Infarct Healing through Reducing Cardiomyocyte Death and Promoting Angiogenesis. Cell Death Dis. 11 (5), 354. 10.1038/s41419-020-2508-y 32393784 PMC7214429

[B109] Wu T. Chen Y. Du Y. Tao J. Li W. Zhou Z. (2018). Circulating Exosomal miR-92b-5p Is a Promising Diagnostic Biomarker of Heart Failure with Reduced Ejection Fraction Patients Hospitalized for Acute Heart Failure. J. Thorac. Dis. 10 (11), 6211–6220. 10.21037/jtd.2018.10.52 30622793 PMC6297406

[B110] Xiao C. Wang K. Xu Y. Hu H. Zhang N. Wang Y. (2018). Transplanted Mesenchymal Stem Cells Reduce Autophagic Flux in Infarcted Hearts via the Exosomal Transfer of miR-125b. Circ. Res. 123 (5), 564–578. 10.1161/circresaha.118.312758 29921652

[B111] Xue R. Tan W. Wu Y. Dong B. Xie Z. Huang P. (2020). Role of Exosomal miRNAs in Heart Failure. Front. Cardiovasc. Med. 7, 592412. 10.3389/fcvm.2020.592412 33392270 PMC7773699

[B112] Yang J. Yu X. Xue F. Li Y. Liu W. Zhang S. (2018). Exosomes Derived from Cardiomyocytes Promote Cardiac Fibrosis via Myocyte-Fibroblast Cross-Talk. Am. J. Transl. Res. 10 (12), 4350–4366. 30662677 PMC6325490

[B113] Yang P. C. (2018). Induced Pluripotent Stem Cell (iPSC)-Derived Exosomes for Precision Medicine in Heart Failure. Circ. Res. 122 (5), 661–663. 10.1161/circresaha.118.312657 29496797 PMC5836744

[B114] Yang T. Martin P. Fogarty B. Brown A. Schurman K. Phipps R. (2015). Exosome Delivered Anticancer Drugs across the Blood-Brain Barrier for Brain Cancer Therapy in *Danio rerio* . Pharm. Res. 32 (6), 2003–2014. 10.1007/s11095-014-1593-y 25609010 PMC4520542

[B115] Zhao J. Li X. Hu J. Chen F. Qiao S. Sun X. (2019). Mesenchymal Stromal Cell-Derived Exosomes Attenuate Myocardial Ischaemia-Reperfusion Injury through miR-182-Regulated Macrophage Polarization. Cardiovasc Res. 115 (7), 1205–1216. 10.1093/cvr/cvz040 30753344 PMC6529919

[B116] Zhou J. Ghoroghi S. Benito-Martin A. Wu H. Unachukwu U. J. Einbond L. S. (2016). Characterization of Induced Pluripotent Stem Cell Microvesicle Genesis, Morphology and Pluripotent Content. Sci. Rep. 6, 19743. 10.1038/srep19743 26797168 PMC4726265

[B117] Zhou S.-s. Jin J.-p. Wang J.-q. Zhang Z.-g. Freedman J. H. Zheng Y. (2018). miRNAS in Cardiovascular Diseases: Potential Biomarkers, Therapeutic Targets and Challenges. Acta Pharmacol. Sin. 39 (7), 1073–1084. 10.1038/aps.2018.30 29877320 PMC6289363

[B118] Zhu D. Li Z. Huang K. Caranasos T. G. Rossi J. S. Cheng K. (2021). Minimally Invasive Delivery of Therapeutic Agents by Hydrogel Injection into the Pericardial Cavity for Cardiac Repair. Nat. Commun. 12 (1), 1412. 10.1038/s41467-021-21682-7 33658506 PMC7930285

